# Bibliographical Notices

**Published:** 1847-06

**Authors:** 


### BIBLIOGRAPHICAL NOTICES.

*Medical Botany; or Descriptions of the more important Plants used in Medicine, with their History, Properties, and Mode of Administration.* By R. Eglesfeld Griffith, M. D., Member of the American Philosophical Society; of the Academy of Natural Sciences of Philadelphia, &c., &c., [with a motto,] with upwards of three hundred illustrations. 8vo. pp. 704. Philadelphia, 1847.

*Illustrations of Medical Botany: consisting of coloured figures of the plants affording the important articles of the Materia Medica, and descriptive letterpress.* By Joseph Carson, M. D., Professor of Materia Medica in the Philadelphia College of Pharmacy; Member of the American Philosophical Society; of the Academy of Natural Sciences of Philadelphia; Fellow of the College of Physicians, &c. The drawings on stone by J. H. Colen. Vol. 1, Fol. pp. 28. Twenty Illustrations. Philadelphia, 1847.

What a change has occurred in the mode in which scientific subjects are taught in the schools at the present day from that which was common twenty or thirty years ago! Dry descriptions unaided by graphic illustrations are now uncommon, and the teaching, in any department of science, which does not address the eye as well as the ear, is properly deemed imperfect. Every thing is now rendered as demonstrable and demonstrative as possible, and it may be safely said, that the medical student has now much greater facilities for obtaining a due insight into an intricate branch of science than he had formerly. Nor ought this to be forgotten when an estimate is made of the present condition of medical education as compared with the past.

Of botanical works treating of general and medical botany, and illustrated by wood-cuts or copperplates, we have had numerous examples. Some of the oldest "*herbals*" are of this character, and in modern times excellent treatises have appeared, both in Europe and in this country, on medical botany more especially. Those of Woodville of England, and of Roques of France, were well adapted to the objects had in view by their authors; whilst those of Barton and Bigelow, on the vegetable materia medica of the United States, were admirable, and are still worthy of all commendation; but they are no longer attainable; and moreover, many excellent and energetic articles from the vegetable kingdom have been added to the lists of the Materia Medica since these works were issued.

It is well, then, that individuals who—all must admit—are fully competent to the task and the occasion—*Arcades ambo*—should have directed their talents and acquirements to the botanical history of those articles of the Materia Medica, which, here and elsewhere, are properly esteemed amongst the most valuable agents employed by the practitioner.

We may, by the way, express our surprise, that amongst the various and often conflicting sentiments to which the Medical Convention recently held in this city gave occasion, nothing—so far as we know—was said by authority as to the importance, to the professional student, of his attaining some knowledge of botany or natural history, either during his office attendance or at an after period. The published sentiments of some of the reformers must have, indeed, been materially modified and mollified, when we find no remarks from them as to the necessity for the candidate for graduation to pass through a curriculum of study equal or approaching to that required in many of the European schools; and which he could not pass through unless such a curriculum were determined upon, and attention to it rigorously enforced.

We confess we should have greatly preferred to see some recommendation to the practitioner, that he should teach, or cause to be taught, to his office student the elements, at least, of botanical and zoological knowledge, rather than that it should have been urged upon him to take care that a candidate for his office instruction should know as much Latin as would enable him to translate or write a prescription. We cannot help regarding it as a solemn farce to pronounce to the world, that the writing of a prescription, in the mode in which it is done universally in this country, implies any knowledge of Latin whatever, seeing that all who understand *technical* terms and the proper *symbols* can accomplish this without difficulty. Let us take any extemporaneous prescription by way of elucidation. We write the following at random :

℞. Infus. Gentian. comp. f.℥vj.

Tinct. Gentian. comp. f.ʒiij.

Syrup Aurant. f.ʒij.

M.

Let him take a fourth part four times a day.

To write such a prescription, we say, requires no knowledge of Latin. The technical titles of the officinal preparations are given in the United States Pharmacopœia; and the abridgement of those titles assuredly demands no Latin. The symbols, too, are understood with equal facility, and with equal nescience of that learned language. It would be somewhat otherwise were the *directions* to be written in Latin. Then, instead of the English, given above, we should have

"Sumat partem quartam quater in die,"

and for this a certain acquaintance with the Latin would be necessary. This, however, is never done in this country, and consequently, we repeat, the writing of a prescription in our ordinary mode requires rather a knowledge of technical terms than of Latin; and hence every apothecary's boy soon learns all that is required of the professional student by the Convention, whether his attention at school have been directed to the humanities or not. We think that respectable body ought to have required—as we stated in a former number—"that the youth intended for the medical profession, should have the intellectual and moral training that befits the well-educated gentleman," and all will be prepared to admit, that the Greek and Latin languages form a necessary part of such training. Were it universal or common we should not meet with such a quotation as the following in a recent number of a respectable medical journal:—*Romæ scribo et Ære romano*"—we give it literally—which "meaneth when literally interpreted"—"I write at Rome and in Roman *brass*," whereas the author wished to say, that he wrote in the "Roman air or atmosphere," *(aere.)* But we must not dwell upon this subject, especially as we do not yet possess a published report of the proceedings of the Convention, and may therefore unintentionally do it injustice.

As regards the utility of natural history and botany, and the importance of their forming a part of medical education "*prior to attendance on lectures*," we borrow and adopt the following observations from a recent author:* "It is not"—he says—"in its relation to materia medica, that the study of natural history ought to be esteemed most important. As physiology investigates the nature and functions of all living bodies, it is, necessarily, intimately associated with natural history. It is, indeed, indebted to this branch of physics more perhaps than to any other. A comparative view of the various gradations amongst organized beings has taught us to appreciate the nature of the several functions that characterize vitality; and has demonstrated, that in proportion as the structure is more complete the functions are more numerous and perfect. Repeated observations, and multiplied experiments on various tribes of animated nature, have elucidated many doubtful and obscure phenomena in the economy of man; and a continuation of this method of research promises to place physiology on the firm basis of rational experience; and to enable us to reason—where only we can reason with safety—by a deduction from facts. The more numerous these facts, and the more satisfactory their arrangement, the more extensive and the more secure will be the foundation they afford for physiological conclusions.

"Botany might seem to be of much more service to the physician than zoology, inasmuch as so many of our remedies are derived from the vegetable kingdom. At one time, indeed, nothing but 'galenicals,' as they were termed, were employed, and these were mainly of vegetable origin. We can imagine the importance of an acquaintance with the botanical characters of different vegetables, should destiny cast the physician on some unknown shore, where the sole sustenance may have to be derived from the vegetable kingdom, and where hundreds, perhaps, may have to be guided to a knowledge of the innoxious and the noxious by his decision. It might happen, too, that the physician may be so situated as to be unable to procure those indigenous productions, which are usually selected so carefully by the professed herbalist as to render it less necessary that they should be culled by him. In such cases his botanical knowledge would be called into play. Still, these are rare emergencies, on

*Dr. Dunglison, Medical Student, p. 165.

account of the facility with which articles of merchandise can be transported everywhere; and, as the preparation of medicinal productions constitutes a distinct calling, the physician is generally in the habit of depending upon the apothecary—who gets them from the herbalist—for his supplies. It is of more practical importance that the physician should know the genuineness of every article that comes to him—a knowledge which observation—rather than botany—gives him. Still, like every branch of the tree of knowledge, physiology and the natural sciences in general have a tendency to expand the mind, and to react upon trains of thought, with which they do not, at first, appear to be intimately associated. In but few of the medical schools of this continent is botany or natural history made a distinct branch of medical education. The period of the year at which medical instruction is chiefly conveyed is unfavorable to botanical exercises; but the seasons of Spring, Summer and Autumn, are well adapted,—especially the first, when all nature smiles; and

> "From the meadow to the wither'd hill,
> Led by the breeze, the vivid verdure runs,
> And swells and deepens; and the spicy groves
> Put forth their buds, unfolding by degrees,
> Till the whole leafy forest stands displayed,
> In full luxuriance, to the sighing gales."

"At these seasons, the student cannot do better—should his opportunities, whilst in the office of his preceptor, permit—than make himself acquainted practically with botany—both by study and observation in the fields; and should he be unable to become a good zoologist,—so far as regards a knowledge of the generic and specific characters of animals,—he can, at least, acquire a knowledge of the 'philosophy of zoology'—one of the most interesting of the applications of natural science, and one that throws important light on the functions of the human body. It embraces, indeed, the physiology of animals, every topic of which elucidates that of man."

The work of Dr. Griffith contains a large amount of instructive matter, and is, in all respects, such a production as might have been expected from one so well acquainted with his subject, and who was for many years a popular teacher on materia medica and other departments of medical science in Virginia and Maryland, and, as a matter of consequence, well aware of the kind of information that ought to be imparted on a congenerous subject. The objects which he was desirous of accomplishing in the work before us, are thus expressed in the preface:

"The work now offered to the profession is intended as a companion to the more practical treatises, and also to convey such information on the systematic classification, characters and history of medical plants as has been necessarily omitted in the publications alluded to. In the execution of the plan, the author has experienced some difficulty in determining the limits of the work. To notice all the plants which have at different times been employed in medicine, or have had remedial properties assigned to them, would have been impossible in the compass of a single volume, and merely to describe those recognized in the Pharmacopœia would have militated against the object of the work. It was thought that the end would be best attained by dwelling at some length on the most important articles of the Vegetable Materia Medica, or on such as are involved in some obscurity as regards their botanical characters and history, and by noticing the others in a brief manner. In doing this, they have been arranged according to the natural orders, and it will be seen that the technical descriptions have been drawn up in strict accordance with the present improved state of botanical knowledge. These descriptions are selected from the best authorities; in some cases without alterations, but in others altered, corrected or condensed, so as to present as great a uniformity of phraseology as possible. As the work is, from its very nature, a compilation, the only originality that can be claimed by the author is in the selection and arrangement of his materials." p. viii.

And is not this the very "originality" that is above all things desirable in such an undertaking; and is not an author deserving of infinitely more honour, who faithfully depicts and interprets science *as it is* than if he *strained* after originality,—with the necessary result of describing science *as it is not*,—and attempted to introduce novelties, which, after myriads—we may say—of excellent observers had bent their minds to the task, must have been noticed by them if the novelty were truth? The "ear is pained, the heart is sick" with the notoriety—fame, perhaps—attained by a naturalist for having first depicted a new species or variety, for which he perhaps deserved but little more credit than he who discriminates amongst a crowd, by his *sensible* properties, one whom he has never before seen, and describes him so well, that he can be readily recognized when seen again by others. Yet upon such *perceptivity*—to employ the language of the phrenologist—the naturalist prides himself; and hosts of his brethren—proverbially *genus irritabile*—are ready to pounce upon him, and to contest the honour or deny the truth of his "*discovery*." The man, who, in the nineteenth century, in a science like that of medicine, collates well and analyses accurately the observations of himself and others, and reduces the *indigesta moles*—the chaos—to something like order, is eminently worthy of honour and of a niche in the temple of fame: it is not, indeed, the best observer of facts who is necessarily the best philosopher—in other words, the best interpreter of the *laws* of facts or phenomena: he serves, however, as the pioneer, and the man of science follows to cultivate the newly discovered region.

To make his work more extensively useful, Dr. Griffith has—wisely, we think—prefixed a brief introduction on the anatomy, physiology and chemistry of plants, with a copious glossary of botanical terms—most valuable to the tyro—and a conspectus of the natural order of plants that are of medicinal utility.

To exhibit the mode in which he treats his subject we shall extract at random.

"I. TINCTORIA, *Linn.*—Leaflets 4—5 pairs, ovate, somewhat pubescent beneath; racemes shorter than the leaves; legumes terete, arcuated, deflexed.

Linn., *Sp. Pl.* 1061; Lam., *Dict.* iii. 245; Lindley, *Fl. Med.* 242; De Candolle, *Prod.* ii. 224.

*Common Name.*—Indigo Plant.

*Foreign Name.*—Indigotier, *Fr.*

*Description.*—A shrub about two feet in height, with spreading, sub-flexuous branches, which are angulose and appressed-puberulous near their extremities. Leaves pinnate. Leaflets 4—5 pair, with an odd one, petiolated, elliptic, acute at base, mucronate at tip, with an appressed pubescence beneath. Stipules small and subulate. Racemes axillary, not as long as the leaves. Flowers pedicellate, with minute subulate bracts. Calyx 5-toothed, the two upper wider apart than the others. Flowers of a greenish colour, marked with vermilion red. Standard ovate, mucronate, minutely ciliate, and pubescent externally. Wings shorter than the keel, which is concave, greenish, minutely, ciliated. Legume more than an inch in length, arcuated, terete, pubescent, containing ten seeds.

Several species of Indigofera are cultivated, but the *I. tinctoria* is that grown in India, and very extensively in South America, the Guatimala plant, *I. disperma*, being considered by De Candolle as a variety of it. Besides this, the *I. anil*, *I. caroliniana*, and *I. argentia*, are also used, the two latter in the United States. The *I. tinctoria* abounds most in colouring principles, and is therefore the one generally selected for cultivation.

Indigo is a rich blue substance, light and friable, tasteless, almost devoid of smell, of a smooth fracture, insoluble in water or alcohol, but dissolved by sulphuric or nitric acid. It consists of indigotin, or indigo blue, indigo brown, indigo red, and a gelatinous substance. It is procured in three different modes; by fermentation, which was the most general plan, by scalding, and by the dry process; which latter is becoming much used in India, and is said to afford the best product, whilst it is not so injurious to the health of those engaged in the manufacture. (See *Encyclop. Amer.*, Art. *Indigo.*) In whatever way Indigo is procured, a certain degree of fermentation appears necessary, as it does not appear to exist in the leaves, and is therefore rather a product than an educt.

The mode of preparing it, and of applying it to the purposes of dyeing, seem to have been known in India from the earliest ages, and it is noticed by Dioscorides and Pliny, though the term *Indicum* was also applied to other colouring substances. Its use was likewise known to the Mexicans and other American natives, long anterior to the conquest. As early as the fifteenth century the Venetians were in the habit of receiving it from India, by the way of Egypt; but it was not generally employed in Europe until about the middle of the sixteenth century, when it was brought from the East Indies by the Dutch. When it was thus introduced, there was a great prejudice against it, and it was considered to be a kind of stone; it was prohibited in England by Elizabeth, and in Saxony by the Elector, who speaks of it in his edict as a corrosive substance, and food fit only for the devil.

The best Indigo is of a deep-blue colour, inclining to violet, of a smooth grain, and bright and sparkling when broken. It should break easily, swim in water, and burn freely, leaving but little residue.

*Medical Uses, &c.*—A decoction of the root, used as a lotion, effectually destroys vermin, and is much used for that purpose in Jamaica. The juice of the young branches, mixed with honey, is recommended as an application in the aphthous sore mouth in children, and Indigo sprinkled over foul ulcers is said to cleanse them (Macfayden, *Flor. Jam.* i. 251.) The leaves are supposed to have virtue in hepatitis, given in the form of powder mixed with honey, and a decoction of the root is reckoned as alexipharmic. (Ainslie, *Mat. Ind.* i. 179.)

Indigo itself has been employed for a long time. The Romans ascribed extraordinary virtues to it; 'rigores et impetus sedat et siccat ulcera,' (*Plin.* lib. xxxv. c. vi.) It was employed at one time as an astringent, in immoderate discharges of the lochia, and for curing a prolapsus of the uterus or rectum. (James, *Pharm. Univer.*, 345.) Of late years it has attained some celebrity in the treatment of spasmodic diseases, especially epilepsy, in which it is stated to have been very successful in numerous cases in Germany. The trials made with it in England and this country, have not been attended with the same good results (Dunglison, *New Rem.*, 361.) To produce any effect the doses must be as large as the stomach can bear, beginning with a few grains and increasing. The best form of exhibition is in an electuary of one part of Indigo to two of syrup. According to Roth (*Pereira*, ii. 610) it produces the following effects. 'Shortly after taking it the patient experiences a sense of constriction at the fauces, and an impression of a metallic taste on the tongue. This is followed by nausea, and frequently by vomiting. In some persons the vomiting is so violent as to prevent any further use of the remedy. When it has subsided, a diarrhœa, often accompanied with cardialgia, ensues; the stools are frequent, liquid, and of a blue colour. Dyspepsia and vertigo sometimes occur. The urine becomes of a dark-brown or violet colour. After its use for some time, spasmodic twitching of the muscles sometimes takes place.'

This article, however, appears to be possessed of very little power, as most persons can take it in very large doses; two ounces having been administered daily for a length of time without producing any very manifest effect, except a derangement of the digestive apparatus."

The wood-cuts are generally well executed; and we can recommend the work strongly both to the student and practitioner.—A few errors we notice—many of them doubtless typographical—such as *Madur Culotropis* for Madar or Mudar Calotropis; *Gieger* for Geiger; Paulus *Æginetus* for Paulus Ægineta. These can be corrected in a second edition, which we hope to herald before a long period has elapsed. The German synonymy, too, requires "overhauling;" in such cases for example as *Neiswurz* for Niesewurz; *Gartenmohu* for Gartenmohn; *Meerettig* for Meerrettig; *Pomeranzin* for Pomeranzen; *Wiesser zimmet* for Weisser Zimmt; *Geftsumach* for Giftsumach; *Unchte Quassie* for Quassie, [we know not what the *unchte* means,] &c. &c. Nor do we think the best or distinctive synonymes have been selected: for example, under Sinapis nigra we have as the German synonym *Schwarzer Senfe*, [Schwarzer Senf] which means "black mustard;" whilst under Sinapis alba, "white mustard," we have *Senfsamen* or "mustard seed," in place of *weisser Senf*. *Bitter wurzel*—"bitter root"—is doubtless a synonym of Gentiana lutea; but the one most used is *Enzian wurzel*, which is omitted. These, however, are comparatively small matters; but we wish to see so good a book purged of all errors. To one who is not a German scholar, or who does not desire to become one, the German synonymy is of course of little or no moment.

The work of Dr. Carson differs from that of Dr. Griffith essentially in this;—that whilst the text is subordinate, in the former, to the "Illustrations,"—in the latter, the xylographic representations are subordinate to the text. This difference between the works is sufficiently shown by a comparison of the title pages.

After stating in his "Introduction" the necessity for a lecturer, who is desirous of being successful, "to have at his command the means of presenting to his hearers the visible objects, or representations of them, about which he discourses," Dr. Carson adds:

"As regards the department of Materia Medica, the instruction usually given is accompanied by the exhibition of delineations of Medical Plants. This will answer the ends of the teacher, and greatly aid the listener for the time being; but amidst the multiplicity of objects, and from the brief period allowed for inspection, the impression made upon the mind is soon enfeebled, and in most cases altogether fades, if close and more protracted observation be not afforded. It is then desirable to possess the means of reviving impressions received, of studying the subject at leisure, and of rendering the plants familiar. But not only to students will the publication be serviceable; it will materially aid numerous teachers, whose facilities of access to the works from which the necessary materials for illustration can be derived, are few and imperfect, and in this respect a double end will be accomplished." * * * "As the design of the work," he concludes, "is simply to present the botanical history of the materia medica, and not a complete account of it, with the exception of indicating the modes of operation peculiar to each substance, all therapeutical and pharmaceutical details appertaining to it have been omitted. The works especially written with the view of unfolding them are full, and easy of access." p. 6.

It is proper to remark, that after acknowledging the sources whence information has been derived, Dr. Carson adds:—

"From the collection of specimens, which during the last ten years he has been enabled to make, and which have been employed by him in the Courses at the Philadelphia College of Pharmacy, many of the representations are entirely new, and where they are not strictly so, corrections have been made from this source, which render them more valuable than those which have been used as copy." p. 6.

The following account of a well known indigenous article of the materia medica will exhibit the Author's mode of managing the text:

POLYGALA SENEGA.—*Linnæus.*

*Seneka Snake Root.*

SEX. SYST.—Diadelphia Octandria.

GEN. CHAR.—*Sepals* five, persistent, the alæ large and petaloid. *Petals* three, their claws all united with the staminiferous tube, the lower one (*carina*) keel-shaped, the two additional ones abortive. *Stamens* united into a tube at the base, which is cleft in front; *anthers* opening by a pore. *Ovary* two-celled; *ovules*, solitary, pendulous from the apex of the cell. *Capsule* two-locular, loculicidal, compressed. *Seeds* pendulous from the apex of the cells, pubescent with a caruncculate arillus at the hilum; *albumen* abundant, fleshy. *Shrubs* or herbaceous plants. *Flowers* arranged in terminal or axillary racemes. (Wright and Arnott in Lindley's *Flora Medica.*)

SPECIF. CHAR.—*Root* perennial, large, firm, and ligneous, with coarse branches. *Stem* nine to fifteen inches high, mostly several from the same root; simple, herbaceous, rather flaccid, and oblique, terete below, slightly angular above; minutely roughish-pubescent, with numerous small, ovate, sessile scales, like leaves, at or near the base. *Leaves* one to two, or three inches long, and one-third of an inch to near an inch wide, smoothish, slightly serulate or scabrous on the margin, more or less acuminately tapering at apex, and narrowed at base to a short petiole. *Spike* one to two inches long, dense, terminal, somewhat nodding, or flaccid; *pedicels* very short, each with an oblong lance-shaped bract at base, and two minute lateral bracts. *Flowers* greenish-white. Capsule obcordate, compressed, orbicular, retuse. *Seeds* large, pyriform, hairy.

This plant is an inhabitant of the United States; found in Pennsylvania, but more abundantly in the Southern and Western States. It flowers from June to August, and ripens its seeds as it flowers.

The root, which is the medicinal portion, is of various sizes, sometimes as thick as large quills, and at others minute and delicate. The head is disposed in the old roots to be enlarged, rough, and irregular, from the separation of the stems annually. It is branched, fibrous, contorted, and twisted, and marked by a sharp line or ridge, which extends the entire length. It is composed of a cortical substance and a ligneous cord. The colour varies from a dark-brown to a yellow. The dried root resembles the fresh, but is broken with a short fracture. It has a peculiar, disagreeable smell, and the taste is at first sweetish but afterward acrid and disagreeable.

In this root have been detected two new acids by Quevenne, *Polygalic acid* and *Virgineic acid*, as also tannic acid. The first is capable of union with bases; it is the principle called by Gehlen *Senegin;* the second is volatile and oily, and may be the volatile oil detected by Dulong. The acrid taste is due to the polygalic acid.

The medical properties of Senega are determined by the dose. In large quantity it is a nauseant and emetic; in smaller, diaphoretic, expectorant, and diuretic. It cannot be regarded as poisonous, although much inconvenience may be induced by an overdose. It is used in pulmonary affections, principally as a stimulant expectorant. It has also been proposed as an emmenagogue.

The introduction to the notice of the medical profession is due to Dr. Tennent, of Virginia, who became acquainted with it from learning that the Indians used it as a remedy in the bites of venomous snakes; hence the name of *Snake-root*. This remedial power, however, has not been sustained. It has been given in powder, but as it imparts its virtues to water, some of the preparations are preferred, as the decoction or syrup. Cox's Hive Syrup owes a part of its properties to this root.

This is only the first part of the work. The Prospectus informs us, that it is to be completed in five numbers, of twenty plates each,—the price of each number to subscribers being five dollars, payable on delivery.

The plates are beautifully executed on stone, and, in the case of plants with which we are familiar, are accurately coloured and life-like. We may infer, therefore, that such is the case with those with which we are unacquainted. Can further recommendations be needed for an undertaking so creditable to all concerned, and which, whilst it instructs the scientific inquirer, must be an object of interest and even of *virtu* to all?

---

Sydenham Society's Works. *On the Injuries and Diseases of Bones. Being Selections from the collected edition of the Clinical Lectures of Baron Dupuytren, Surgeon-in-chief to the Hôtel-Dieu at Paris. Translated and edited* by F. Le Gros Clark, Assistant Surgeon to, and Lecturer on Descriptive and Surgical Anatomy at St. Thomas's Hospital. 8vo. pp. 459. London: 1847.

This is the second work issued by the Sydenham Society of London to its members of the year 1846—7. The first, containing Hewson's contributions to scientific medicine, was noticed by us in a late number.

At the request of the Council of the Society, Mr. Clark undertook to translate and edit a volume, which should comprise all the papers on the Injuries and Diseases of the Bones contained in the *Leçons Orales* of the most distinguished French surgeon of modern times; and the following introductory remarks will explain the "scope, objects and arrangement of the work."

"In the original," says Mr. Clark, "the articles in question are scattered through the first two volumes of the 'Leçons Orales,' without any regard to appropriate collocation. Moreover, much confusion is the consequence of frequent and sudden changes from the first to the third person;—the Professor being made sometimes to speak in the former, at others in the latter; or, again, his editors speak for him, or quote his opinions. In the translation it has been thought desirable to impart an uniform style to the work; as well as to render the French into corresponding idiomatic English, as far as was compatible with a strict adherence to the meaning of the author. To carry out this intention, a paraphrastic translation has been, in a measure, rendered essential. Compression, also, has been resorted to in details generally; and many of the cases have been abridged, especially in those parts of them which have no immediate reference to the subjects under consideration. In short, the object has been, to render the present treatise more acceptable to the English reader, by disencumbering the original of needless repetition and redundancy,—a process of condensation to which one cannot but believe that the author himself would have subjected the work, had he superintended its publication in a complete form; although it is not difficult to appreciate the motives of his able editors, in retaining and enlarging upon all that fell from their esteemed preceptor." p. vi.

A biographical sketch of the author is prefixed to the volume, which has the rare merit, that whilst it depicts in glowing terms the elevated intellectual qualities of the subject of the biography, it does not conceal his admitted failings. It is a picture of the man as he was, not as the biographer would wish him to have been.

The third issue of the Sydenham Society for the year will be the works of the illustrious Harvey.

---

*Lectures on subjects connected with Clinical Medicine; comprising Diseases of the Heart.* By P. M. LATHAM, M. D., Fellow of the Royal College of Physicians, Physician Extraordinary to the Queen, and late Physician to St. Bartholomew's Hospital. 8vo. pp. 365. Ed. Barrington and Geo. D. Haswell. Philadelphia, 1847.

In his capacity of physician to one of the largest Hospitals in London, during a number of years, the author of these lectures enjoyed unusual opportunities for watching the phenomena and progress of disease, and the influence of medical treatment, and few men have evinced greater zeal to avail themselves of such advantages, or have been endowed with higher powers for profiting by them. Unlike many of our most admired investigators of the present day, he does not present us with a mass of heterogeneous matter in the shape of facts, without the least seeming object or combination; but resemblances are noticed, concurrences pointed out, and deductions, natural and obvious, are drawn, so that the reader is made to comprehend the philosophy as well as the facts of the science.

The object of the lecturer has been to present to the student a concise account of the diseases of the heart, as met with at the bed side, and not that extended investigation into all that relates to the subject, which is found in the more comprehensive work of Dr. Hope. It differs, too, from the latter work in strictly avoiding all controversy. "Mine," he remarks, "is a limited purpose. It is to regard the diseases of the heart only in one point of view, i. e., as they appear in the living man. But this one point of view includes the several objects of their clinical diagnosis, and their clinical history, and their medical treatment." Rightly conceiving that "the clinical diagnosis of diseases of the heart, owes all the higher degrees of certainty to which it has been carried in our own times, entirely to auscultatory signs," "the natural sounds and impulses and resonances" of the organ are accurately described, in order that the degree and extent of variations from these may be understood as evidences of unsoundness. The whole subject, of diseases of the heart, including angina pectoris and pericardiac affections, is treated in a brief but masterly manner.

---

*A treatise on the diseases of the eye.* By W. Lawrence, F.R.S., Surgeon Extraordinary to the Queen, etc. etc. etc. *A new edition. Edited, with numerous additions, and one hundred and seventy-six illustrations,* by Isaac Hays, M. D., Surgeon to Wills' Hospital; Physician to the Philadelphia Orphan Asylum; Member of the American Philosophical Society; Fellow of the College of Physicians of Philadelphia, etc. etc. 8vo. pp. 859. Lea and Blanchard, Philadelphia, 1847.

The treatise on diseases of the eye by Mr. Lawrence has been long before the profession, and is too extensively, and, we may add, too favourably known to require lengthened notice or particular commendation. It commences as, in our opinion, all such treatises designed for the use of students and general practitioners ought to commence, with a full account of the anatomy and physiology of the eye-ball. After describing the various parts in the normal condition, and their functions in health, the lesions both of structure and function are more readily discovered and better appreciated. Such an introductory course is indispensable to the student, and to the country practitioner, who has not access to extensive treatises on anatomy and physiology, scarcely less so.

The author's descriptions of the various diseases of the eye, lachrymal organs, &c., are remarkably full and explicit, and from his large experience and thorough reading, there can be little doubt, that the treatment pointed out in the several affections is in accordance with the present advanced state of our knowledge in this important branch of surgical science.

"Among the additions which have been made," says the Editor, "may be noticed,—the descriptions of several affections not treated of in the original—an account of the catoptric examination of the eye, and of its employment as a means of diagnosis—one hundred and seventy-six illustrations, some of them from original drawings,—and a very full index."

The editor has had much experience in the treatment of this class of diseases, in private practice as well as in public institutions, and his contributions therefore constitute a valuable part of the publication. We cannot but regret, however, the evident haste in which these contributions have been written. Had he allowed himself more time in the preparation of his notes and commentaries, he would doubtless have corrected and rendered more perspicuous many passages which are now obscure; and, we hope, would have more generally followed the nomenclature adopted in the U. S. Pharmacopœia. He might have written strych*nine* for strych*nia*; but certainly not carbonate of potassi*um* for carbonate of potass*a*; hydriodate of potassi*um* for hydriodate of potass*a*,—*iodide of potassium;* &c. We should have been pleased, also, to have discovered more frequent reference to contemporaneous American writers on diseases of the eye, and a less exclusive reliance on the pages of a single American Medical Journal (Am. Jour. of Med. Science) for cases and opinions. Still, we can most heartily commend this edition of Lawrence on the Eye, as a rich store-house of ophthalmic knowledge.

